# Pulmonary tuberculosis mimicking cystic metastases: evolution from multiple cystic cavities to nodules during therapy

**DOI:** 10.1186/s12879-026-13809-2

**Published:** 2026-06-13

**Authors:** Chengqing Yang, Chunlin Mei, Shufang Chen, Minhui Mei, Chao Quan, Xuan Wang

**Affiliations:** Wuhan Pulmonary Hospital (Wuhan Institute for Tuberculosis Control), Hubei Branch (Wuhan Pulmonary Hospital) of the National Clinical Research Center for Infectious Diseases, No. 28 Baofeng Road, Qiaokou District, Wuhan, Hubei Province People’s Republic of China

**Keywords:** Pulmonary tuberculosis, Cystic lung disease, Cystic cavity, Nodules, Atypical radiology

## Abstract

Pulmonary tuberculosis typically exhibits characteristic imaging findings; however, radiological manifestations can be atypical in certain cases, leading to potential misdiagnosis based solely on CT imaging. We describe a 59-year-old man who developed a 4-month history of cough and a 2-week fever during postoperative radiotherapy following esophagectomy for esophageal cancer. CT scans revealed multiple, scattered cystic cavities in both lungs, characterized by regular shape and well-defined borders. Following anti-tuberculosis therapy, the pre-existing cystic cavities collapsed and evolved into nodular opacities, a change highly suggestive of cystic metastases given the patient’s history of esophageal cancer. A biopsy confirmed granulomatous inflammation, and follow-up at eight months after the initiation of anti-tuberculosis therapy showed near-complete resolution of the lesions. Although cystic pulmonary tuberculosis is rarely reported, this case is notable for its distinct cystic characteristics. To our knowledge, this is the first case to systematically document the transformation of cystic lesions into nodules during anti-tuberculosis therapy—a finding that may raise suspicion for cystic metastases and highlights the diverse and atypical radiological manifestations of tuberculosis in immunocompromised hosts.

## Introduction

Pulmonary Langerhans cell histiocytosis (PLCH), Birt-Hogg-Dubé syndrome (BHD), lymphangioleiomyomatosis (LAM), Pneumocystis jirovecii pneumonia (PJP), and lymphoid interstitial pneumonia (LIP) are well-recognized causes of cystic lung disease [1–4]. Beyond these classic entities, a range of extremely rare etiologies can also manifest with pulmonary cysts, including light-chain deposition disease [5], cystic pulmonary amyloidosis [6], metastatic malignancy [7], and infectious diseases such as tuberculosis [8]. Pulmonary tuberculosis presenting with multiple cystic cavities is exceptionally rare, with only a few case reports describing this imaging feature. The radiological evolution of cystic cavities collapsing into nodules during anti-tuberculosis therapy is even rarer. We report a case of an immunocompromised patient with esophageal carcinoma who developed multiple bilateral cystic pulmonary cavities secondary to tuberculosis, raising suspicion for cystic metastases. During anti-tuberculosis therapy, these cavities collapsed and evolved into nodular opacities. This case expands our understanding of the spectrum of atypical radiological responses in tuberculosis.

## Case report

**Fig. 1:**
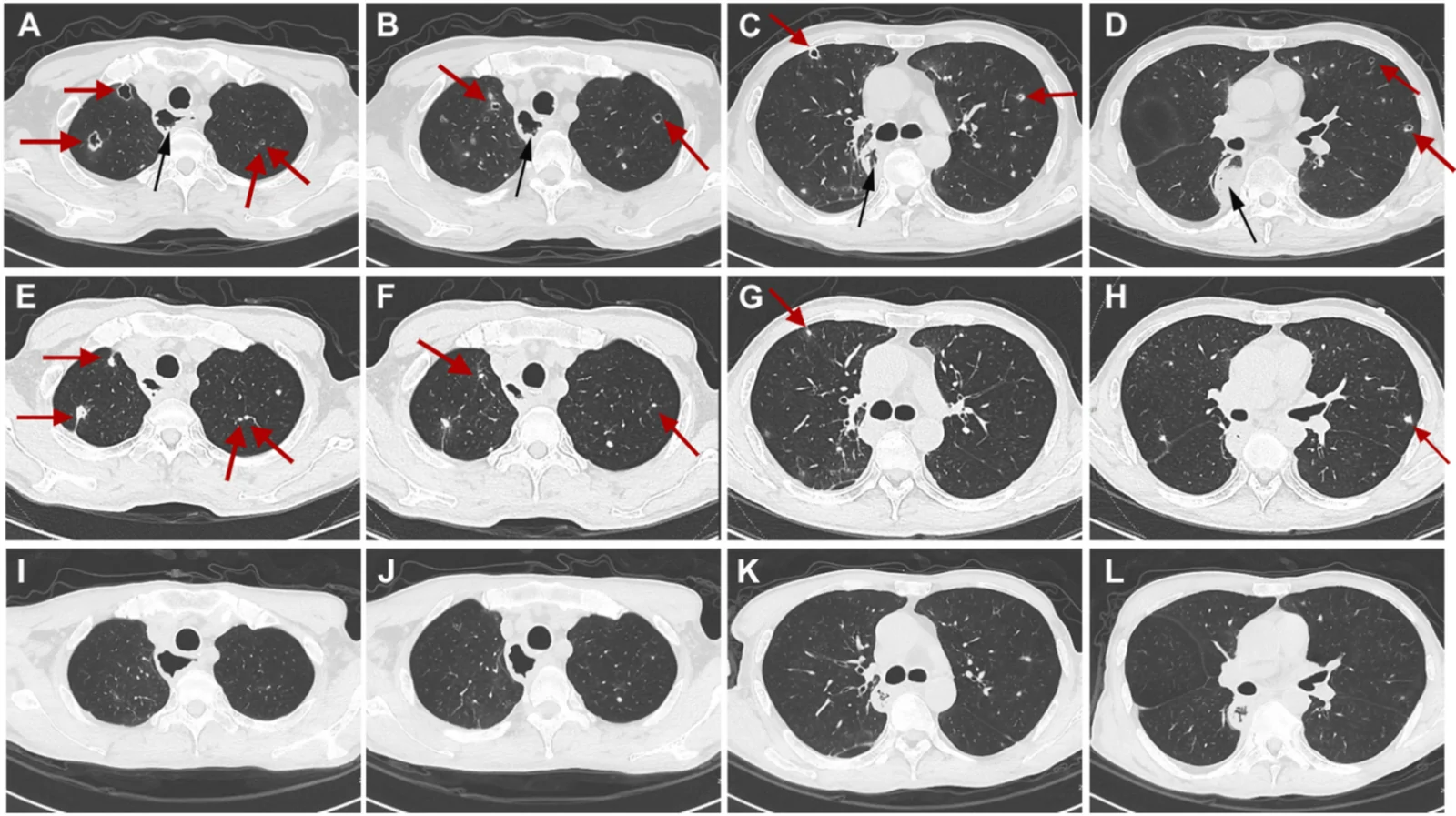
Chest CT findings in a patient with cystic pulmonary tuberculosis before and after treatment. (**A**-**D**) Pre-treatment CT images (lung window) reveal multiple bilateral thin-walled cystic cavities (red arrows) with scattered micronodules, predominantly in the upper lobes. (**E**-**H**) CT images at 2 months following the initiation of anti-tuberculosis therapy (ATT) demonstrate the transformation of the cystic cavities into coalescent nodules (red arrows) at corresponding anatomical levels. (**I**-**L**) CT images at 8 months of ATT show near-complete resolution of the previously noted lesions. Note: Images **A**, **E**, **I**; **B**, **F**, **J**; **C**, **G**, **K**; **D**, **H**, **L** represent sequential scans at the same anatomical levels, respectively. A post-esophagectomy gastric pull-through is also noted (black arrows in **A**–**D**)

A 59-year-old male with diabetes mellitus and hepatitis C, who had undergone esophagectomy followed by radiotherapy for esophageal cancer, presented with a 4-month history of cough and a 2-week history of fever. He had received conventional antibacterial antibiotics (e.g., cephalosporins) at an outside hospital, but the fever persisted despite treatment. Preoperative chest computed tomography (CT) revealed no significant pulmonary abnormalities. Upon admission, chest high-resolution CT (HRCT) demonstrated multiple, scattered cystic cavities in both lungs with regular shape and well-defined borders. The cystic cavities measured 1–15 mm in diameter, with wall thickness predominantly ranging from 1 to 2 mm; in only one or two cavities did the wall thickness reach up to 4 mm. These lesions were predominantly located in the upper lobes of both lungs, accompanied by diffusely scattered micronodules (Fig. 1A-D, arrows).The patient denied any history of smoking or environmental or occupational exposure to dust or other inhalational hazards. Laboratory findings indicated pancytopenia (WBC 2.58 × 10⁹/L, Hb 115 g/L, PLT 121 × 10⁹/L), elevated inflammatory markers (hsCRP 6.59 mg/L, ESR 29 mm/h), and a normal plasma D-dimer level (410 µg/L). Serum tumor markers including carcinoembryonic antigen (CEA), squamous cell carcinoma antigen (SCC), and cytokeratin 19 fragment (CYFRA21-1) were all within normal limits. T-SPOT.TB was positive, hepatitis C virus antibody was positive, and HIV antibody was negative. Analysis of bronchoalveolar lavage fluid (BALF) from the apical segment of the right upper lobe yielded negative results for acid-fast bacilli staining, routine bacterial culture, fungal culture, galactomannan assay, Aspergillus nucleic acid, Pneumocystis jirovecii nucleic acid, and Candida nucleic acid. However, Xpert MTB/RIF testing detected *Mycobacterium tuberculosis* (MTB), and metagenomic next generation sequencing (mNGS) of the BALF identified 31,329 sequence reads of MTB. Cytological examination revealed no malignant cells. A preliminary diagnosis of pulmonary tuberculosis was made, and standard anti-tuberculosis therapy (ATT) was initiated with a 2HRZE/6HRE regimen (Isoniazid 300 mg, Rifampicin 450 mg, Pyrazinamide 1500 mg, and Ethambutol 750 mg daily for 2 months, followed by Isoniazid 300 mg, Rifampicin 450 mg, and Ethambutol 750 mg daily for 6 months). Concurrently, the patient received insulin therapy (Insulin NovoRapid 30, 10 IU in the morning and 8 IU in the evening) for glycemic control and Bicyclol (25 mg three times daily) for prophylaxis against drug-induced liver injury, given his underlying chronic hepatitis C. Subsequently, mycobacterial culture results were positive, indicating drug susceptibility to first-line anti-tuberculosis agents.

**Fig. 2:**
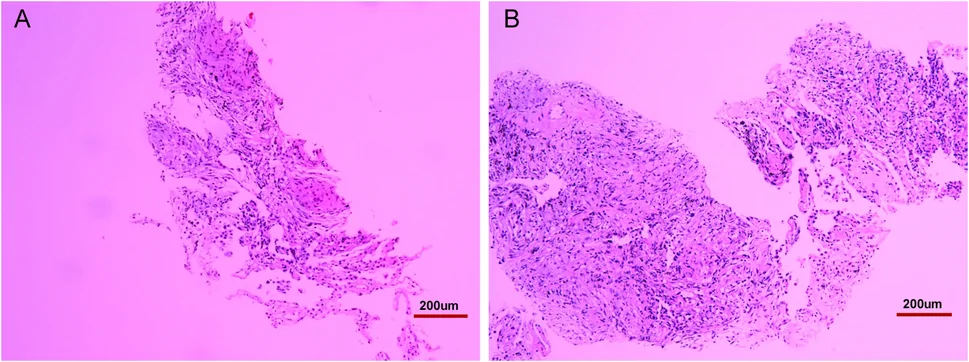
Histopathological confirmation of non-necrotizing granulomatous inflammation. (**A**) Hematoxylin and eosin staining of the CT-guided lung biopsy specimen shows well-demarcated granulomas composed of epithelioid histiocytes, without necrotic centers. (**B**) Lung tissue demonstrates architectural destruction with proliferating fibrous connective tissue containing multiple small, ill-defined non-necrotizing granulomas

After 2 months of anti-tuberculosis therapy, a follow-up CT showed that the cystic cavities had collapsed into coalescent nodules (Fig. 1E–H, arrows). To clarify the nature of these newly formed nodules, a CT-guided biopsy of the nodule in the apical segment of the right upper lobe, which had transformed from a cystic cavity, revealed non‑necrotizing granulomatous inflammation (Fig. 2). Mycobacterial culture of the biopsy tissue was negative, but tissue TB-DNA was positive, thus excluding metastatic tumor. Eight months later, the patient’s symptoms had resolved, and imaging showed near-complete clearance (Fig. 1I–L). A detailed clinical timeline is presented in Table 1.

**Table 1:** Clinical timeline of the case

Date/Time	Clinical Events
Before esophagectomy ( about 5 months before admission)	Chest CT performed 4 days prior to surgery showed no significant abnormalities; the patient was prepared for esophagectomy with gastric pull-up reconstruction.
During postoperative radiotherapy (about 4 months before admission)	Cough developed during radiotherapy.
Outside hospital (15 days before admission)	Presented with fever; no response to ceftazidime treatment.
Day 1 of admission	1. Cough for 4 months and fever for 2 weeks.
	2. HRCT: multiple, scattered, cystic cavities in both lungs with regular shape and well-defined borders.
	3. Past history: Hepatitis C, diabetes mellitus, post-esophagectomy.
	4. Laboratory tests: Pancytopenia, elevated hsCRP and ESR.
	5. T-SPOT.TB positive; HCV antibody positive.
Day 2 of admission	Bronchoalveolar lavage (apical right upper lobe): Xpert MTB/RIF positive; mNGS positive for MTB (31,329 reads); other microbiological and cytological tests negative.
Day 3 of admission	Standard anti-tuberculosis treatment initiated: 2HRZE/6HRE regimen.
2-month follow-up	1.CT showed cyst-to-nodule transformation at corresponding sites.
	2.CT-guided biopsy of the cyst-to-nodule transformation site: granulomatous inflammation; tissue TB-PCR positive.
8-month follow-up	CT showed near-complete resolution of lesions; patient asymptomatic.

## Discussion and conclusions

In this case, HRCT revealed multiple, bilateral, scattered, thin-walled (mostly 1–2 mm) cystic cavities with smooth inner margins and well-defined borders, accompanied by micronodules. Pulmonary tuberculosis presenting predominantly with multiple cystic cavities is extremely rare. Wang et al. [8] reported a case of tuberculous infection with imaging showing diffuse, bilateral, thin-walled cystic lesions of varying sizes, accompanied by consolidation. Kodati et al. and Churako et al. [9, 10] respectively reported diffuse cystic lung disease due to tuberculosis in immunocompetent patients, with cystic cavities of varying sizes and diverse morphologies, alongside focal consolidation or spontaneous pneumothorax. In comparison, the cystic cavities observed in the present case were more regular in shape, relatively fewer in number, more discrete, and without associated consolidation or pneumothorax, suggesting that tuberculosis-related cystic lung disease may present with diverse imaging patterns.

In the differential diagnosis, septic pulmonary embolism (SPE) was excluded based on negative blood cultures, a normal transthoracic echocardiogram, and a normal D-dimer level. Mycobacterial blood culture, urine culture, and bone marrow biopsy were not performed; therefore, clinical evidence for miliary tuberculosis remains insufficient, although it cannot be completely excluded. Although other diffuse cystic lung diseases, such as PLCH, BHD, and LAM, were included in the differential list, they were not prioritized as diagnostic considerations given the primary concern for metastatic disease in this clinical context. Given the patient’s history of esophageal carcinoma, cystic pulmonary metastases were the foremost clinical consideration. The literature indicates that various malignant tumors can manifest as cystic or cavitary metastases, with proposed mechanisms including the “check-valve” effect due to tumor growth along the bronchiolar walls, central ischemic necrosis of the lesion, and tumor regression following treatment [11]. Tsuji et al. [12] reported a case of cavitary lung metastasis after esophagectomy for esophageal cancer; the distinction from our case is that theirs presented as a solitary thick-walled cavity, whereas our case presented with multiple cystic cavities (wall thickness 1–2 mm). Tiresse et al. [13] reported a case of metastatic lung adenocarcinoma initially presenting as diffuse bilateral pseudo-cystic pulmonary lesions, with CT showing diffuse bilateral air-filled lesions with thin, occasionally irregular walls measuring less than 2 mm in thickness—a case highly similar to ours. These reports suggest that diffuse cystic lesions in patients with known malignancy do not allow for complete exclusion of a malignant etiology. However, in the present case, malignancy was systematically excluded through multimodal diagnostic approaches: serum tumor markers (CEA, SCC, CYFRA21-1) were normal, BALF cytology revealed no malignant cells, CT-guided lung biopsy confirmed non-necrotizing granulomatous inflammation with positive tissue TB-DNA, and the lesions resolved under anti-tuberculosis therapy, thereby confirming tuberculosis.

Several mechanisms have been proposed to explain the formation of tuberculous cystic/cavitary lesions. The most widely accepted hypothesis is that tuberculous granulomas involving the small airways create a “check-valve” effect, leading to air trapping and cystic dilation of the distal alveoli, ultimately resulting in the formation of thin-walled cystic cavities [14, 15]. With effective anti-tuberculosis therapy, the mycobacterial burden in the bronchial lumen decreases and granulomatous inflammation subsides, allowing absorption of the air within the cyst and passive collapse of the cystic cavity [16]. Subsequently, the host immune response drives the influx of macrophages and epithelioid cells, which along with lymphocytes form non-necrotizing granulomatous nodules, filling the collapsed cystic space [17]. However, the precise molecular mechanisms driving the cyst-to-nodule transformation remain to be elucidated.

In conclusion, this case highlights the atypical radiological manifestations of pulmonary tuberculosis in immunocompromised patients. It underscores that, even when imaging findings raise strong suspicion for metastatic malignancy in the context of esophageal cancer, a high index of suspicion for tuberculosis should be maintained, and comprehensive multimodal diagnostic confirmation—including CT-guided biopsy with molecular testing—should be pursued. The cyst-to-nodule transformation observed during therapy represents a host immune response and reparative process.

## Data Availability

No datasets were generated or analysed during the current study.
